# Correction: Micheli et al. Beneficial Effect of H_2_S-Releasing Molecules in an In Vitro Model of Sarcopenia: Relevance of Glucoraphanin. *Int. J. Mol. Sci.* 2022, *23*, 5955

**DOI:** 10.3390/ijms26104476

**Published:** 2025-05-08

**Authors:** Laura Micheli, Emma Mitidieri, Carlotta Turnaturi, Domenico Vanacore, Clara Ciampi, Elena Lucarini, Giuseppe Cirino, Carla Ghelardini, Raffaella Sorrentino, Lorenzo Di Cesare Mannelli, Roberta d’Emmanuele di Villa Bianca

**Affiliations:** 1 Department of Neuroscience, Psychology, Drug Research and Child Health-Neurofarba—Section of Pharmacology and Toxicology, University of Florence, 50139 Florence, Italy; laura.micheli@unifi.it (L.M.); clara.ciampi@stud.unifi.it (C.C.); elena.lucarini@unifi.it (E.L.); carla.ghelardini@unifi.it (C.G.); 2 Department of Pharmacy, School of Medicine and Surgery, University of Naples Federico II, 80131 Naples, Italy; emma.mitidieri@unina.it (E.M.); carlotta.turnaturi@unina.it (C.T.); domenico.vanacore@unina.it (D.V.); cirino@unina.it (G.C.); demmanue@unina.it (R.d.d.V.B.); 3 Department of Molecular Medicine and Medical Biotechnology, School of Medicine, University of Naples Federico II, 80131 Naples, Italy; raffaella.sorrentino@unina.it

In the original publication [1], there was a mistake in Figure 5 as published. During the assembly of the representative panel, the condition “dexamethasone + glucoraphanin” of carbonylated proteins and the house-keeping protein β-Actin were unintentionally represented by wrong images. The corrected Figure 5 appears below.

The authors state that the scientific conclusions are unaffected. This correction was approved by the academic editor. The original publication has also been updated.

## Figures and Tables

**Figure 5:**
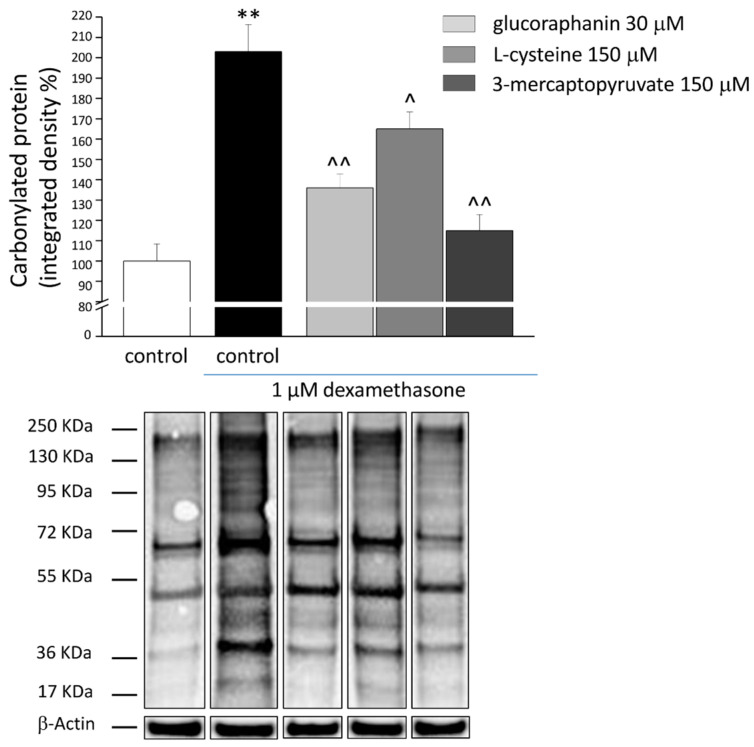
Protein carbonylation in C2C12 myotubes. C2C12 myotubes were treated for 48 h with 1 μM DEX, both alone and in the presence of 30 μM glucoraphanin, 150 μM L-cysteine and 150 μM mercaptopyruvate. Western blot analysis was carried out on cell homogenates using a specific antibody against DNPH. Figures show the densitometric analysis (**top**) and the illustrative immunoblot (**bottom**). Each sample was normalized to β-Actin expression. Values are expressed % of integrated density and calculated as mean ± S.E.M. of 3 different experiments. ** *p* < 0.01 versus control; ^ *p* < 0.05 and ^^ *p* < 0.01 versus DEX.
